# Autonomy, Evidence and Methods in Global Health

**DOI:** 10.1007/978-3-030-62662-4_2

**Published:** 2020-11-24

**Authors:** Louise Ackers, Gavin Ackers-Johnson, Joanne Welsh, Daniel Kibombo, Samuel Opio

**Affiliations:** 6 Global Social Justice, University of Salford, Salford, UK; 7 University of Salford, Salford, UK; 8 Infectious Disease Institute, Kampala, Uganda; 9 Pharmaceutical Society of Uganda, Kampala, Uganda

**Keywords:** Antimicrobial resistance, Antimicrobial stewardship, Infection Prevention Control, International development, Global health, Maternal sepsis, Pharmaceuticalisation, Ethnography

## Abstract

This chapter discusses the growing impact that funding bodies have on the design, delivery and evaluation of global health interventions with specific emphasis on the UK’s **Commonwealth Partnerships for Antimicrobial Stewardship** (CwPAMS) funding programme. It explains the reasons for focusing the antimicrobial resistance intervention on maternal sepsis and describes the context within which the Maternal Sepsis Intervention took place; in a Regional Referral Hospital in Western Uganda.

This chapter addresses a key question: how do we create a high quality, Fit-For-Purpose, evidence base for global health interventions that optimally combines ‘change’ objectives with the generation of credible, scientific evidence?

Evidence in International Development, as in many policy domains, has played two rather different roles. On the one hand, it concerns the quest for an evidence base to guide policy. On the other, it represents a response to an increasingly cynical political environment, in an age of austerity, that questions the efficacy of public expenditure on Aid; the ‘giant cashpoint in the sky’.^1^ Evaluation, as the generation of knowledge, then merges with financial and political accountability. Moyo captures this concern poignantly when she argues that Aid is malignant, and evaluation has contributed to a smoke screen:

In nearly all cases, short-term aid evaluations give the erroneous impression of aid’s success […] The notion that aid can alleviate systemic poverty, and has done so, is a myth. (2010: xix)

Rajkotia (2018) makes a similar point referring to the enormous pressure on global health institutions and the foreign aid ‘industry’ to achieve targets. This he suggests leads to a tendency to ‘embellish’ reporting and, worse still, fabricate or overattribute achievements (p. 1). Storeng and Palmer (2019) detail how the pressure on donors to be seen to deliver on investments can contribute to serious challenges to researcher independence and even censorship of results. This, they argue, contributes to ‘tick-box evaluations designed to please donors’ (p. 185).

The emphasis on evaluation rather than research in funding calls is indicative of this merging of two rather uncomfortable bedfellows; financial accountability and knowledge. It lies behind the emergence of an entirely new cadre of impact assessment ‘experts’ and evaluators typically juggling identities as project managers/evaluators. The link with accountability mechanisms has, perhaps unintentionally, centre-staged a specific approach to evaluation that has been the subject of substantial critique for over 50 years in academic research. The term ‘paradigm’ is often used to describe the domination of a specific way of thinking that shapes attitudes and behaviour. And the paradigm we are referring to here has been known as ‘ positivism’.^2^ Positivist methods—and the emphasis on measurable (quantitative) outcomes or ‘ac/counting’—may meet the needs for financial accountability (or Value For Money: VfM). But is it the best approach to knowledge generation and transfer in health systems research?

Bridget Somekh traces the parallel development of increasingly radical social science theories and a policy context (the politics of sponsored research) that has, ‘*moved in the other direction and is ideologically framed now in more totalitarian assumptions of traditional research practices than was the case in the 1970s and 1980s*’ (2006: 5). Somekh goes on to critique the positivist paradigm and, with specific reference to research on education systems in the UK, explains how this has led to an emphasis on technical solutions:

… we are locked in unrealistic assumptions of the application of natural science research methods to social situations; there is a belief in a process of incremental knowledge building to construct a technology of definite [educational] solutions for generalised application across contexts. (p. 5)

Harding makes a similar point in the context of international development critiquing not only the emphasis on training (a point we return to) but also the underlying methods and conclusions they infer:

The transfer of Western scientific rationality and technical expertise from the West to “the rest” had always been the “motor” of modernisation theory and now drives development policy. However, many of the assumptions about women and poor people in the Global South - were false. (2015: 152)

The point about generalisability is important in the current context. One of the reasons for the emphasis on ‘measurable outcomes’ amongst international organisations, such as UKAid or the WHO lies in the perceived need to aggregate outcomes from diverse interventions to demonstrate cumulative impact (and benchmark change over time). How does an organisation such as the UK’s Department for International Development (DfID), with a budget of £14 billion and under huge public scrutiny, capture impacts across a plethora of interventions spanning the scope of the Sustainable Development Goals (from health, gender equality, economic growth etc.)? How do we compare the efficacy of a project on cervical cancer screening in Malawi with one on childhood disability in Sudan?

It would be hard to question the merits of this goal and understand the resort to metrics. But will those metrics become so generic that we focus on the measurable at the expense of the meaningful?

This is often the challenge facing not only end of the line project implementors and researchers but also intermediary funding organisations.^3^

Quick fix technical approaches based on highly unreliable and ‘sanitised’ secondary data are more likely to caricature reality than they are to capture genuine change and the costs associated with that. Harding, in a seminal feminist critique of research methods, refers to the use of evaluators in such situations as ‘fast guns for hire’ who can relay one version of events about the world ‘ready-made for reporting’ [but] without listening to women’s accounts’ (1991: 158). With reference to the claim to greater objectivity that underpins the positivist approach, Harding makes a very critical and relevant assertion:

Paradoxically, the more “scientific” social research becomes, the less objective it becomes. (1991: 140)

The concept of objectivity is associated with notions of bias; it is based on the idea that there is a single truth that exists outside of any investigation, and the job of the researcher or evaluator is to avoid contamination of the data (facts). The concept of subjectivity, on the other hand, conveys the idea that a researcher is a person who interacts with the world and the people they are studying and their values and experiences inevitably shape the ‘data’ they generate. People have values that impact the way they see the world and influence ‘truth’ claims. Stephen Jay Gould points to the fundamental subjectivity of science:

Science, since people must do it, is a socially embedded activity. It progresses by hunch, vision, and intuition […] the most creative theories are often imaginative visions imposed upon facts; the source of imagination is also strongly cultural. (1981: 303)

These are complex philosophical concepts and we do not attempt to explore them in depth here; rather to illustrate their impact on the evidence base guiding international development in general and the conditions framing our antimicrobial resistance project in Uganda. In a field so explicitly and necessarily focused on change and with very powerful normative drivers (or value commitments) to universal health coverage, gender equality and empowerment—the approach to data and objectivity associated with positivism is simply untenable. These substantive value commitments that applicants for international development funding are required to (and should) align themselves to represent an immediate challenge to conventional ideas of objectivity and the ‘d’ words identified as synonyms of objectivity: disinterest; dispassion; detachment.

Harding argues that, ‘*It is a mistake to assume that research shaped by social values and interests invariably will be empirically unreliable. Maximal objectivity and a commitment to a more democratic organisation of the research process need not conflict… they can often enhance each other*’ (2015: 151).

The concept of partnership working embraced in the Sustainable Development Goals and echoed in UKAid policies also presents serious challenges and lies in genuine and significant tension with the deductive principles underlying positivism. This is particularly problematic with short term, ‘hit-the-ground-running’ approaches to funding where applicants are required to present a highly specified ‘theory of change’^4^ at application stage. Even where scoping work^5^ has been undertaken, outlining a theory of change at the start of a complex project on antimicrobial resistance is precisely the kind of deductive approach that fails to meet the principles of partnership outlined in SDG 17. The Health Partnership Scheme managed by the Tropical Health and Education Trust has developed principles of partnership that all applicants are required to align themselves to.^6^ These include ‘listening to one another; cultivating trust; proactively adapting to change and aligning interventions to national planning’ and suggest the need for a far more iterative, responsive and relational approach to both intervention and research than is possible within a positivist, theory-testing framework. The ideas of listening to each other resonates with Harding’s description of research as an ‘*affirmation of ordinary life*’ (1991: 158).

We would argue that the specific combination of change objectives with evaluation (research) predicated in most Official Development Assistance (ODA) work are best captured through the principles of Action Research. Action research is typically associated with more inductive approaches to theory generation; rather than starting with theories and trying to test them (somewhere else) ‘in the field’, action research is driven by contextual dynamics. Although researchers and actors will come to projects with prior knowledge (and in that sense cannot approach any social situation with an entirely blank slate) theory generation in projects will emerge informed by the context, ongoing review of other work and through active inter-personal relationships (partnership engagement). Somekh describes, ‘*… the ways in which social science researchers use action research methodology to overcome the limitations of traditional methodologies when researching changing situations. Action research combines research into substantive issues […] with research into the process of development in order to deepen understanding of the enablers of, and barriers to, change. It is a means whereby research can become systematic intervention, going beyond describing, analysing, and theorising social practices to working in* *partnership* *with participants to reconstruct and transform those practices. It promotes equality between researchers from outside the site of practice and practitioner*-*researchers from inside, working together with the aspiration to carry out research as professionals, with skilful and reflexive methods and ethical sensitivity*’ (2006: 1).

Perhaps, with the exception of doctoral research, most research is conducted in partnership with funding bodies who have their own objectives. This inevitably shapes our ability to pursue the ‘sociological imagination’ (Wright-Mills 1959) and achieve optimal reflexivity. Harding acknowledges the need for compromise and recognition that other values will enter the negotiation process with funding bodies and experts:

Scientists must balance their interests with those of their funders and sponsors even if one thinks they don’t do so as vigorously as the anti-authoritarian and citizens science movements have been demanding. Scientists also negotiate routinely with experts of other fields with whom their research requires collaboration […] negotiating such relations is what social life is about including the social life of science. (2015: 167)

Whilst we accept the need for balance, the result of this blurring of evidence with accountability has been for increasingly prescriptive funding calls to reduce project autonomy. This limits the scope for critical reflexivity and meaningful contextualisation. There is also a serious risk that high-level project management, by increasingly ‘engaged’ funding bodies, becomes a strait jacket with outcome measures framing interventions rather than permitting iterative, intelligent, approaches to influence outcomes. In effect, the tail may be increasingly wagging the dog to the detriment of knowledge and social change.

The specific nature of the funding stream as described above reflects the values of the parties involved, their approach to international engagement in general and AMR in particular. These have shaped the way the Maternal Sepsis Intervention has evolved often through ‘creative tension’. We hope that by being explicit about this we can contribute to the co-production of more effective approaches to improve the evidence-base in global health. Achieving optimal objectivity in social research demands humility, honesty and the exercise of caution when making evidence claims. This requires openness about the objectives of funders, the normative underpinnings of these and the impacts these have on project design, implementation and outcomes.

The following section outlines the Call for Funding and the objectives and approaches applicants were invited to align themselves in order to position themselves for funding. The detail is presented here as an illustration of the level of complexity and prescription that shapes many, if not most, international development programs.

## The Commonwealth Partnerships for Antimicrobial Stewardship (CwPAMS) Programme

 The Commonwealth Partnerships for Antimicrobial Stewardship (CwPAMS) is a partnership involving three very different organisations: The UK Department of Health and Social Care, the Commonwealth Pharmacists Association and the Tropical Health and Education Trust. CwPAMS was awarded £1.3 million as part of a, ‘wider commitment by the UK Government to spend up to £265 million of UK aid to support LMICs to enhance their surveillance of AMR by 2021’.^7^ The Department of Health and Social Care manages this ambitious UKAid programme through the Fleming Fund. Although the Fleming Fund takes an holistic approach, there is a strong underlying emphasis on capturing ‘surveillance’ data to show patterns of resistance to antimicrobials (or which antibiotics are no longer effective in fighting infections). Despite the very serious and immediate threat that AMR poses to global health, there is still very little understanding of international patterns of resistance especially in LMICs where the capacity to generate and use surveillance data is particularly weak and uneven. Ultimately capturing resistance patterns, globally, represents the best approach to assessing the phenomenon of AMR; its responsiveness to environments (such as COVID-19) and interventions designed to contain it. This underlying emphasis on surveillance of resistance is expressed quite succinctly:

The aim of the Fleming Fund is to get data relevant to AMR in the hands of decision makers. We want to support countries generating the data they need to inform policies and practices which will optimise the use of antimicrobial medicines.^8^

The Fleming Fund programme is made up of Country and Regional Grants and a Fellowships programme (administered by the Management Agent Mott MacDonald) and a variety of Global Projects managed directly by DHSC of which CwPAMs is one. The parent Fleming Fund includes extensive independent evaluation with an emphasis on:

- how much the quantity and/or quality of data on AMR at country level has increased, and to what extent the Fleming Fund has contributed to this increase
- to what extent the Fleming Fund’s investments have been aligned with other relevant investments at country level
- how sustainable the country level data quantity and/or quality increase is likely to be
- whether improved AMR data has influenced (a) changes in national policies/regulations, and/or (b) changes in practice and attitudes in each country
- how much the quality of data shared and reported internationally has improved, and whether the Fleming Fund has contributed to this
- whether the Fleming Fund’s investments at country level offer value for money

The Tropical Health and Education Trust (THET) is a UK-registered charity focused on health system strengthening in LMICs through, ‘ training and educating health workers in Africa and Asia, working in partnership with organisations and volunteers from across the UK’.^9^ THET was appointed as operational partner for the CwPAMS programme and allocated the funds through its established and prestigious ‘Health Partnership’ model. Twelve new and established Health Partnerships across four African countries (Ghana, Uganda, Tanzania, and Zambia) shared £600,000 of the £1.3 million in direct project funding.^10^ Through, ‘regular short-term visits’ the partnerships were designed to, ‘leverage the expertise of UK health institutions and technical experts to strengthen the capacity of the national health workforce and institutions to address **predefined** AMR challenges’.

 The Commonwealth Pharmacists Association (CPA), on the other hand, is focused on Commonwealth countries and on improving the quality of pharmacy practice.^11^ The CPA have acted as a technical partner to THET on CwPAMS. Their role includes carrying out a scoping analysis, developing or providing assistance in the development of AMS resources, supporting grant holders with bespoke advice in AMS, and developing and analysing programme-level AMS reporting tools and data.

The predefined challenges referred to above were aligned to three of the Fleming Fund Objectives, with a specific focus on antimicrobial stewardship and the *use* of antimicrobials.^12^

###  Fleming Fund Priorities Identified by CwPAMS

- Support the development of National Action Plans for AMR
- **Developing and supporting the implementation of protocols and guidance for AMR surveillance and antimicrobial use.**
- Building laboratory capacity for diagnosis
- Collecting drug resistance data
- Enabling the sharing of drug resistance data locally, regionally, and internationally
- **Collating and analysing data on the scale and use of antimicrobial medicines**
- **Advocating for the application of data to promote the rational use of antimicrobials**
- Shaping a sustainable system for AMR surveillance and data sharing
- Supporting fellowships to provide strong national leadership in addressing AMR

The emphasis is on quite programmatic features with a strong assumption that LMICs need support in developing protocols and implementing these. Secondly, an emphasis on data and specifically antimicrobial consumption data. Finally, a strong normative assumption that this data will form the basis of effective advocacy for more rational use of antimicrobials. The Guidance to potential applicants added further complexity identifying three ‘Themes’; with a *requirement* for each applicant to address Themes 1 and 2 (but not 3):

***CwPAMS ‘Themes’***

1. Antimicrobial stewardship, including surveillance – requirement!
2. Antimicrobial pharmacy expertise and capacity – requirement!
3. Infection Prevention Control

Theme 1 reinforces the emphasis on stewardship recognising the relationships between stewardship (use of antimicrobials) and surveillance (of resistance patterns). Theme 2 adds a specific twist on this immediately identifying the role of pharmacy as the key discipline in antimicrobial use. The Guidelines then specified several outcomes:

***CwPAMS Outcome Specifications***

- Institutions and workforce demonstrate improved knowledge and practice related to AMS prescribing practice and IPC
- Evidence of effective AMR interventions, with standardised tools and guidance being used by local institutions and/or national partners
- NHS staff demonstrate improved leadership skills and a better understanding of the global context of AMR in their work

It is interesting to note that, although IPC was not a required ‘theme’, outcomes in relation to IPC were listed.^13^ Outcome 1 anticipates both a knowledge premium (the ability to evidence **knowledge acquisition** amongst LMIC health workers) and improved practice (**knowledge application** or utilisation) with a very specific emphasis on ‘*prescribing* practice’. Echoing the Fleming Fund Objectives, Outcome 2 specifies a focus on the development of **standardised tools.** The emphasis on ‘standardised’ tools stands in some tension with the commitment to ensure contextual compatibility which lies at the heart of effective implementation. Whilst alignment with national or even international standards and explicit recognition of where such alignment is not possible is important, a ‘one-size fits all’ approach to something as complex as AMR is unlikely to be successful.

Outcome 3 draws on the Department of Health and Social Care’s commitment to building the **expertise of the UK health workforce** (through volunteering). This introduces a welcome bilateral component to mutual learning but infers a difference in the type of knowledge to be acquired by the UK nationals and LMIC health workers. Whilst the knowledge premium for LMIC staff was anticipated in prescribing practice and IPC; the emphasis in UK volunteer learning was on leadership skills and an understanding of AMR in global context.

Later in the same guidance document Project Outcomes are re-stated but this time with a new emphasis on *microbiology* *data* which was not highlighted in the Fleming Fund Objectives. And IPC is very much present in this ‘expectation’.

## Outcomes Expected from Projects in the CwPAMS Call^14^

### What Outcomes Are Expected from You Through CwPAMS?

*Partnerships should strengthen workforce in:*

- Antimicrobial prescribing practice
- Use of microbiology data to inform decision making
- Infection Prevention Control
- Antimicrobial stewardship including surveillance of antimicrobial use

A further set of ‘Scoping Requirements’ placed a strong emphasis on ‘antimicrobial consumption and behavioural drivers of inappropriate use’. This emphasis on antibiotic consumption and the behaviour of individual prescribers could be interpreted as falling within what Denyer Willis and Chandler (2018) characterise as a ‘pharmaceuticalisation’ model centred on pharmaceutical distribution and individual behaviour change. There is a strong emphasis in the ‘Scoping Requirements’ on the role of clinical pharmacy:

Project Activities should, ‘*Build on initiatives in the 4 countries for* *knowledge* *transfer and bidirectional learning to develop AMS as part of the* ***clinical pharmacy*** *role.*’

Importantly, the final ‘Scoping Requirement’ specifies a particular leadership model:

Multidisciplinary team **led/co-led by pharmacists** that model best practice of multi-disciplinary working, especially nurses, pharmacists and doctors working equally.

**Fig. 2.1:**
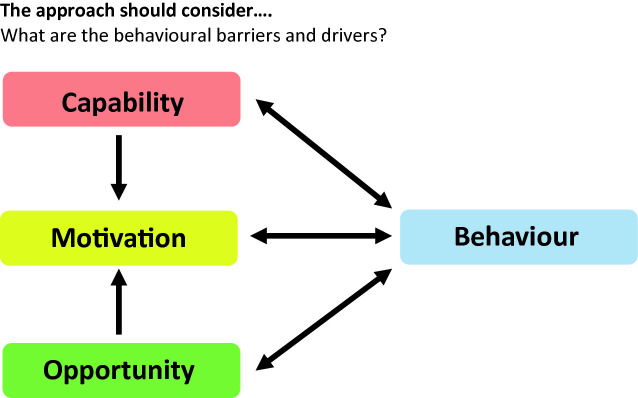
Behaviour change theory in the CwPAMS programme (*Source* Adapted from Commonwealth Partnerships for Antimicrobial Stewardship Call for Applications Webinar)

Behaviour change was also high on the CwPAMS agenda with an emphasis on individual behaviour change as conceptualised in the Com-B Framework approach (Michie et al. 2011). And behaviour change scientists were enabled to join some of the funded projects to support implementation of this approach (Fig. 2.1).^15^

**Fig. 2.2:**
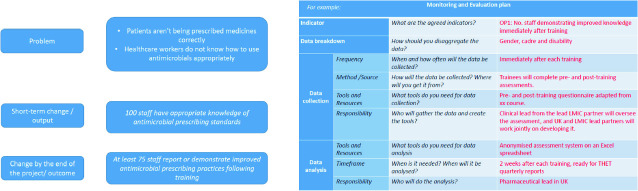
Guidance on assessment of training (*Source* Commonwealth Partnerships for Antimicrobial Stewardship Call for Applications Webinar)

The Guidance also presented applicants with possible models of intervention and associated evaluation mechanisms with a powerful emphasis on ‘measurable’ outcomes. Figure 2.2 illustrate the anticipated/preferred approach and the emphasis on pre and post ‘training’ assessments.

It is important to note that the diagram presented above was for ‘guidance’ purposes only. However, successful applicants are required to complete a ‘logframe’ reporting template. UKAid has produced guidance on log frames and describes them as a multi-purpose tool combining ‘regular project monitoring with annual review processes, project completion reports and evaluation’.^16^ The description in the UKAid guidance of the ‘results chain’ as a ‘logical (linear)’ tool and subsequent reference to ‘objective’ measurement; the necessity of baseline data, attribution, and verification all point in the direction of what Denyer Willis and Chandler refer to as a ‘counting’ approach (2019: 2).

Although the guidance on log frames acknowledges the value of qualitative data it does so within an implicitly deductive, linear-planning perspective. The logic of the logframe methodology is expressed in Table 2.1 designed to capture the outcomes of the CwPAMS project.

**Table 2.1:** Excerpt from CwPAMS logframe

Output 1	*Output indicator 1.1*
LMIC healthcare workforce strengthened in areas of AMS and antimicrobial prescribing practice	No. of LMIC healthcare staff trained in AMS, antimicrobial prescribing practise and consumption surveillance (based on WHO competency framework)
	*Output indicator 1.2*
	No. of LMIC healthcare staff trained and tested demonstrating improved knowledge after training
	*Output indicator 1.3*
	No. and % of LMIC healthcare staff able to demonstrate how to practise their new knowledge

*Source* Commonwealth Partnerships for Antimicrobial Stewardship Call for Applications Webinar

Securing funding is a highly competitive process and potential applicants would ignore the guidance offered by funding bodies at their peril (Storeng and Palmer 2019). It is within this context that the project team designed their application for funding. To put the ambitious goals in context, the scheme launched on October 31st, 2018 with a submission deadline of January 4th, 2019. Grants were due to commence in February 2019. In practice, funding^17^ was allocated in April 2019 with completion due on April 30th, 2020.

Whilst the value positions of funders will steer project design, the project team may be heavily influenced by recent research findings or alignment with their own partnership objectives and expertise. Research and interventions are often cumulative building on pre-existing work and relationships. Indeed, continuity is listed as one of THET’s principles of partnership. The Kabarole Health Partnership (KHP), by way of example, had developed a strong area of expertise and associated relationships in maternal health and were acutely aware of the mortality associated with maternal sepsis. This introduces yet more complexity and ‘steer’ into the project planning process.

## Why Maternal Sepsis?

A recent review of research on antibiotic stewardship (Cox et al. 2017) found limited evidence of effective and feasible stewardship interventions in (LMICs) and, where examples of effective interventions were identified, emphasized the essential need for contextualization. From a hospital management and health worker perspective, outcomes focused on stewardship and antibiotic consumption do not immediately align with urgent and tangible service priorities. A key priority for Fort Portal Regional Referral Hospital^18^ in 2018 was to reduce maternal mortality. As a Health Partnership, we were acutely aware of this priority and need.

Data from the Ministry of Health’s most recent analysis of maternal and perinatal deaths (MOH 2019) indicate that, in the Financial Year 2018/2019, there were 1,180,321 deliveries in health facilities and 1083 maternal deaths. The majority of these deaths were reported from General (472) and Regional Referral Hospitals (334) and, of these, Fort Portal Regional Referral Hospital reported the second highest maternal mortality rate (Table 2.2).

**Table 2.2:**
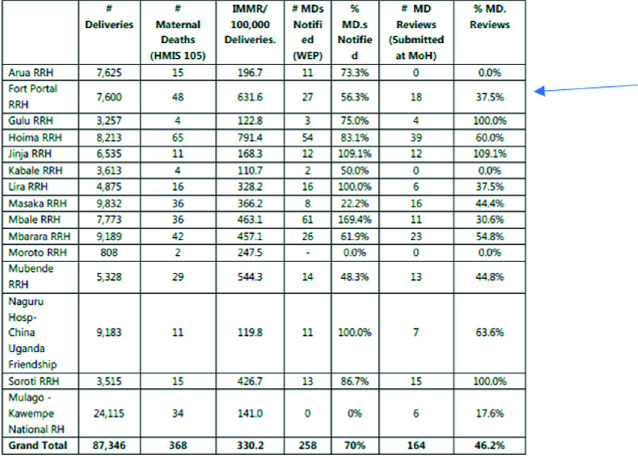
Notification, reporting and reviews of maternal deaths at regional and national referral hospitals

*Source* The National Annual Maternal and Perinatal Death Surveillance and Response (MPDSR) Report FY 2018/2019, Ministry of Health Uganda (September 2019)

According to this report, obstetric haemorrhage remains the leading cause of maternal deaths in Uganda accounting for 46% of all maternal deaths reported, followed by Infections/Anaemia/HIV & other conditions not related to Pregnancy (13%) and hypertensive disorders (11%). The 2019 MPDSR Report found that:

Institutional maternal mortality ratios are highest at the regional and national referral hospitals (RRHs) (382/100,000 deliveries). This could be as a result of late and critical referrals from lower facilities, over-stretched resources (human, financial, equipment), inadequate essential supplies like blood and lifesaving commodities and the delays to access services at the referral sites. The institutions also received high numbers of patients in critical (near death) conditions. (2019: 24)

Ngonzi et al.’s study (2016) in Mbarara Hospital, Uganda reports puerperal sepsis^19^ as the most frequent cause of maternal mortality responsible for 30.9% deaths as compared to obstetric haemorrhage (at 21.6%). Most cases of sepsis following childbirth can be characterised as Surgical Site Infections arising as a direct result of medical intervention (caesarean-section). A project focus on stewardship in relation to maternal sepsis had numerous attractions:

1. Sepsis is a major cause of maternal mortality
2. Sepsis is highly preventable through improved Infection Prevention- Control
3. Sepsis is associated with very high antibiotic consumption

The proposal is built on pre-existing work by the Kabarole Health Partnership (KHP). KHP engages a range of stakeholders in Uganda and the UK including Knowledge For Change (K4C), an NGO registered in the UK and Uganda; the Universities of Salford and Mountains of the Moon; Kabarole Health District and Fort Portal Regional Referral Hospital (FPRRH). K4C functions in an operational role implementing projects on the ground and has a strong local presence in facilities. K4C’s continuous presence on the ground and growing recognition of our principles of partnership and co-presence has led to strong relationships. In order to extend existing relationships to embrace pharmacy and ensure active engagement with the Ugandan National Action Planning process, the partnership expanded to include joint leadership with the Secretary General of the Pharmaceutical Society of Uganda (PSU) and the NAP Team. We also brought in pharmacy expertise from the University of Salford, linking directly to the non-medical prescribing programme^20^ and the lead AMR pharmacist in Tameside and Glossop Hospital Trust.

Based on prior experience, the team proposed a ‘Complex Intervention,’ whole-systems, approach that built on pre-existing knowledge and explicitly allowed for flexibility in response to local contextual dynamics. McCormack describes the dual focus of action research (AR), combining the quest for new knowledge with the goal of achieving social change, as a reason for considering AR in the implementation of complex interventions (2015: 300). He further argues that much of the complexity in complex interventions arises from the context within which any evidence is to be implemented and, citing Bates, suggests that, ‘nothing exists, and therefore can be understood, in isolation from its context’ (2014: 3). The following section outlines the context within which the MSI developed.

## Study Context: The Post-natal and Gynaecology Wards at FPRRH

**Fig. 2.3:**
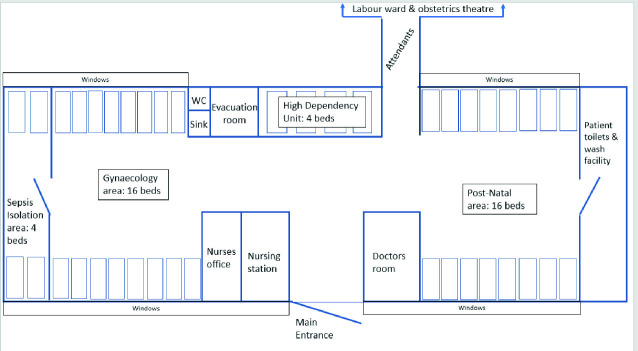
Site plan of the post-natal and gynaecology wards at FPRRH

The Kabarole Health Partnership (KHP) had an active presence on the maternity wards at Fort Portal Regional Referral Hospital. There are two postnatal wards in FPRRH. One is designated for women who have had vaginal births, the other for those who have had caesarean sections. Our focus was on the women on the caesarean section post-natal ward. If these women recovering from c-sections become unwell, they are transferred to the adjoining gynaecology ward or High Dependency Unit. Suspected sepsis cases are managed in an isolation area at the far end of the ward as indicated on the site plan (Fig. 2.3).

The public maternity wards at FPRRH have an average of 800 deliveries per month with a c-section rate of around 20% (160/month). The adjoining post-natal and gynae wards have 40 beds. It is common to have between five and ten floor cases at any one time. There are six nursing officers and five midwives staffing the wards on three shifts with two on duty at any time. Three Senior doctors are employed to work on maternity as a whole: two medical officers and two intern doctors.

## The Intervention

The intervention can best be described as an exploratory ‘journey’ navigated by a multi-disciplinary co-working team. K4C employs Ugandan health workers to support its activities in health facilities; with the same strict guidelines applied to professional volunteers, to ensure co-present working and guard against labour substitution. Two K4C midwives were already working in labour ward. At the start of the project, we relocated these two midwives to post-natal and gynae (PNG). They were joined initially by a UK junior doctor and several months later, a nurse from the NHS with specialist experience in wound care.^21^ This team began to establish close relationships with staff on the wards and across the hospital to understand the context and observe and discuss the management of sepsis. This process stimulated an immediate focus on Infection-Prevention-Control as a preliminary to all other activity. The journey is traced in more detail in the following chapters.

Within the quite prescriptive framework of the CwPAMS funding, the priorities of the hospital and the learning gained from initial scoping work, the team re-defined its objective as follows:

How can we improve antimicrobial stewardship in a Ugandan public referral hospital in a way that improves patient outcomes (in this case associated with maternal sepsis) and demonstrates sustainability through cost effectiveness?

## Methods Used in the Maternal Sepsis Intervention

**Fig. 2.4:**
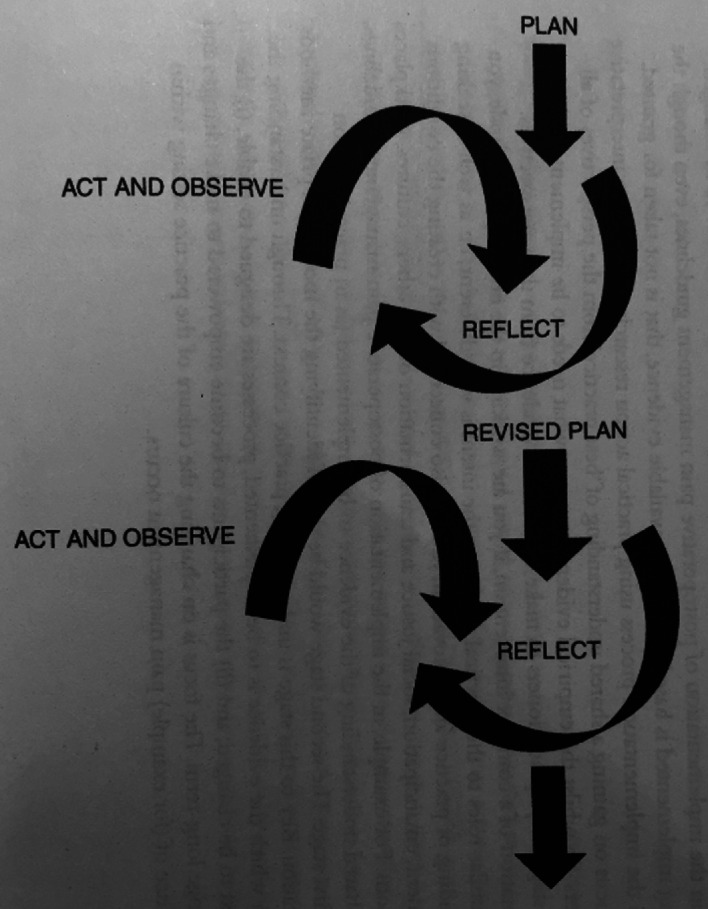
An example of action-research cycles (*Source* McCormack [2015: 303])

 Action research moves away from being *on* people (as objects) to being research that is participatory, *with* people and *for* people (Reason, 1988 as cited by Meyer 1993). Such an approach aligns with THET’s Principles of Partnership. We noted that action research embraces the need for agency from all participants (Meyer 2000). This inclusive approach encouraged and supported individuals to make their own unique contribution to the change process, which in turn promoted group cohesion and the development of relationships between local health care workers and with overseas colleagues. Furthermore, using action research allowed us to move away from the didactic approach to learning that is commonplace in LMIC settings. In action research, the intervention moves together with the research in an iterative and reflexive process. McCormack (2015) describes this as an example of action-research ‘cycles’ with an action triggering a phase of research which then leads to the next action and so forth (Fig. 2.4).

Whilst this is a very helpful way of understanding action research, the idea of defined cycles with a clear starting point and closure is an over-simplification of the messiness of research (Hantrais 2009).

Mutale et al. (2016) emphasise the value of systems thinking in complex interventions in LMICs with specific attention to the generation of unintended consequences, interactions and interdependencies. They critique what they term reductionist approaches that attempt to ‘dissect a complex process and study the individual parts’ (p. 112). Systems theorists engaged in complex interventions, they suggest, prefer a ‘general science of wholeness addressing structures, patterns and cycles in systems rather than seeing only specific events’ (p. 112). Although the language they use is different, the approach proposed resonates with that adopted in this action research intervention.

Our methods can best be described in terms of a multi-method ethnography, commencing, as always, with participating observational work on the ground. Observational work was undertaken on a co-researching basis with a lead role played by K4C staff and volunteers, supported by repeated and extended site visits by the Principal Investigator and virtual co-presence over a 15-month period. The team were joined by the Ugandan lead and attended Hospital IPC meetings on two occasions. Observations complemented by ongoing WhatsApp and Skype conversations were recorded in notebooks, minutes, reports, and emails and entered into NVivo12^22^ for storage and analysis.

This observational research generated theory inductively which, in turn, stimulated the search for other sources of data and honed the focus. Although we had anticipated accessing facility data on antibiotic consumption, we could not have known or understood the complexity of this process and the challenges of even defining consumption in a public hospital setting prior to the start of the project. In such situations and given the essentially inductive quality of ethnographic research, simple a priori (deductive) hypothesis setting is inappropriate. In that respect, a process of conceptualisation, theory generation and data collection took place simultaneously. Every attempt to record or collate data stimulated intense ongoing discussions about the recording processes and the nuances of its interpretation. In most cases, it led us to new lines of enquiry (theories) and approaches to data collection. Much of the data, as is normal in this context, was not collated and had to be manually and painstakingly searched for from casefiles or records books. Files were often missing or incomplete. The very poor quality of documentation in patient files and subsequent records management is a critical dimension of context with implications for AMR. Allegranzi et al.’s systematic review of health-care-associated infections in LMICS notes the lack of data and poor quality of many studies contributing to what they term the ‘hidden and serious burden on health systems and patients’ (2011: 236). Study quality in their sample was related to very poor-quality record keeping and documentation as a result of ‘inaccuracy of information from patients’ records, and a paucity of electronic records or databases for surveillance of health-care-associated infection’ (p. 235).

Data collection became a process of exploration, involving forms of local capacity-building along the way on methods of organising and storing hospital records and entering them into excel spreadsheets. In this context (as in many others), much of the facility-based data could not be interpreted at face value as facts; but rather artefacts reflecting their (social) construction. Facility data has been collected from a wide range of sources. Firstly, data on drug orders and supplies from National Medical Stores (NMS), was obtained through an online national pharmacy database, known as the Rx system, the use of which was functionalised through the project. This was supplemented by data from paper-based records (the Dispensing Log) of supplies distributed from the central hospital stores to the wards. Further, the hospital laboratory, itself supported by the Infectious Diseases Institute (IDI), proved a key partner both in the intervention itself, with laboratory results providing a critical stimulus to multi-disciplinary team working, but also in generating research data. This commenced prior to the project as part of Ackers-Johnson’s microbiology doctorate (Ackers-Johnson 2020) and has continued throughout, generating valuable data on resistance patterns. The laboratory provided data on test results of samples taken from the PNG wards in 2019.

Whilst we would contend that facility-based data at FPRRH cannot be understood as ‘facts’ but rather social constructs contributing to a partial truth, this is not the case with the microbiology (surveillance) data generated under stringent laboratory controls. Although human error can affect the accuracy of this form of data; it is not relational in the same way as facility data. The objective status of the microbiology test results has played a powerful role in breaching disciplinary hierarchies and promoting effective team-working (see below).

This complemented a data set generated from 142 cases of suspected sepsis between January 2019 and February 2020 that were identified through a manual search of paper-based patient records. In January 2020, a phase of qualitative interviewing took place to capture perceptions of the impact and effectiveness of the intervention. Twenty-five interviews were conducted with all cadres involved in the MSI, including 50% of the nurses, midwives, intern doctors, laboratory technicians and pharmacists working on the PNG wards, two hospital managers and three UK volunteers. The interviews were transcribed and thematically analysed using NVivo 12. Ethical approval for the work was gained from the University of Salford, Makerere University, and the Ugandan National Council.^23^

Somekh suggests that not only is the action research process a continual one, it never naturally ends until a decision is taken to take stock and publish its outcomes ‘to date’ (2006: 6). Once again this has clear resonance with the emphasis on continuity that we feel is a feature of the principles of partnership in international development research. Although work has and will continue, this book represents the situation at the end of the initial funding period.

The following chapter presents key outcomes arising from the Maternal Sepsis Intervention and the methods outlined above.

## Footnotes

1 This was a comment made by UK Prime Minster Boris Johnson on 16 June 2020 to support the decision to merge the Department for International Development with the Foreign Office. https://www.bbc.co.uk/news/uk-politics-53062858.

2 Put very simply positivism is an approach developed in the natural sciences which is based on the idea that facts exist (and can be mathematically verified) through scientific experimentation. It is also associated with deductive approaches and theory or hypothesis-testing. According to this approach, a researcher starts with a theory or hypothesis about a phenomenon or social problem and designs experiments to test that. The emphasis on ‘facts’ (as opposed to knowledge) sees the data collector as necessarily external to this process—and as a potential pollutant.

3 This aggregation process has many ‘layers’. At program level, the CwPAMS can be viewed as an intermediary organisation reporting to higher level funders. In that respect, the first phase aggregation process seeks to align projects within a broadly similar framework (described below) albeit in very different contexts.

4 The ‘theory of change’ approach whilst echoing the language of academic research uses the concept of theory in a quite specific way (see Vogel 2012).

5 Scoping visits are often of 1–2 weeks’ duration and, at best, help to establish teams but not to understand context in any meaningful way.

6 https://www.thet.org/principles-of-partnership/.

7 https://www.thet.org/our-work/grants/cwpams/.

8 https://www.flemingfund.org/about-us/.

9 https://www.thet.org/about-us/what-we-do/.

10 The CwPAMS programme is subject to an additional evaluation managed by THET. In terms of VfM measures, the outcomes associated with the 12 projects sharing the £600,000 would be expected to demonstrate VfM for the full £1.3 million placing quite significant pressure on individual projects.

11 https://commonwealthpharmacy.org/.

12 Antimicrobials is a wider term referring to medicines that are active against microbes in general—including fungal and viral infections. The project has focused on bacterial infections and the use of antibiotics to treat those.

13 CwPAMS hoped to embrace IPC through the programme as a whole but not necessarily in every project.

14 These outputs were again expected across the programme and not necessarily within each project.

15 It is important to note that the COM-B Framework is only one approach to behaviour change theory (Ackers and Ackers-Johnson 2016).

16 https://www.ukaiddirect.org/wp-content/uploads/2016/04/UKAD-Guidance-Logframes.pdf.

17 12 projects were awarded between £30,000 and £75,000 each; the MSI received £60,000.

18 FPRRH is often referred to locally as ‘Buhinga Hospital’.

19 Relating to the period up to 6 weeks after childbirth.

20 Non-medical prescribing is possibly one of the most inappropriately named task-shifting programs. In practice, non-medical prescribing means prescribing by non-medical cadres and is actively supported in the UK through an accredited training programme; https://www.rcn.org.uk/get-help/rcn-advice/non-medical-prescribers. No such programmes exist in Uganda.

21 Part of the iterative quality of interventions involving deployment of UK volunteers is contingent upon recruitment processes; the characteristics and timing of volunteer stays inevitably shapes interventions. The lack of lead time in this project, as is usual, restricts the ability to plan in advance. Despite major efforts, we were unable to recruit a long-term pharmacy volunteer for the first 12 months and then the volunteer was unable to travel out due to COVID-19.

22 NVivo is a qualitative data analysis computer software package produced by QSR International.

23 HS249ES.
